# The relationship between climate change induced natural disasters and selected nutrition outcomes: a case of cyclone Idai, Zimbabwe

**DOI:** 10.1186/s40795-023-00679-z

**Published:** 2023-01-27

**Authors:** Vimbainashe Prisca Dembedza, Prosper Chopera, Jacob Mapara, Nomalanga Mpofu-Hamadziripi, George Kembo, Lesley Macheka

**Affiliations:** 1 Centre for Innovation and Industrialisation, Marondera University of Agricultural Sciences and Technology, P.O Box 35, Marondera, Zimbabwe; 2 Department of Nutrition, Dietetics and Food Sciences, University of Zimbabwe, P.O Box MP 167, Mt Pleasant, Harare, Zimbabwe; 3 Institute of Lifelong Learning & Development Studies, Chinhoyi University of Technology, Private Bag 7724, Chinhoyi, Zimbabwe; 4 Institute of Teaching and Learning, Marondera University of Agricultural Sciences and Technology, P.O Box 35, Marondera, Zimbabwe; 5 Food and Nutrition Council, 1574 Alpes Road Hatcliffe, Harare, Zimbabwe

**Keywords:** Climate change, Natural disasters, Nutrition outcomes, Cyclone Idai, Zimbabwe

## Abstract

**Background:**

The increased frequency of climate induced natural disasters has exacerbated the risks of malnutrition in the already vulnerable regions. This study was aimed at exploring the effects of Cyclone Idai on nutrition outcomes of women of child-bearing age and children under 5 years.

**Method:**

The household-based cross-sectional study was conducted in Eastern Zimbabwe. Data were collected through face-to-face interviews to determine food consumption score (FCS) and household dietary diversity (HDDS), minimum dietary diversity for women (MDD-W) and minimum dietary diversity for children (MDD-C). Severity of Cyclone Idai was grouped into five categories based on the extent of damage to infrastructure and loss of human lives. Association between continuous and categorical variables was tested using Pearson correlation test and Chi square test, respectively. Linear and binary logistic regression was performed to investigate determinants of food security.

**Results:**

A total of 535 households were interviewed. There was a significant correlation between severity of Cyclone Idai and MDD-W (*p* = 0.011), HDDS (*p* = 0.018) and FCS (*p* = 0.001). However, severity of Cyclone Idai was not a determinant of any nutrition outcome, but gender of household head was a negative predictor of HDDS (β = − 0.734, *p* = 0.040), and marital status of household head was a positive predictor (β = 0.093, *p* = 0.016) of FCS.

**Conclusion:**

The findings provide a good baseline to inform future programming of food aid activities during disasters. More so, our findings call for evidence-based policies regarding composition of a food aid basket and targeting of beneficiaries. The main strength of this study is that it is the first to investigate the effects of cyclones on food and nutrition security indicators and is based on a large sample size thus making our results generalisable.

## Introduction

Climate induced natural disasters such as floods, cyclones, storms, and heat waves have been on the rise globally and have doubled since the early 1990s [1]. These disasters are now becoming more frequent and extreme due to an increase in greenhouse gases concentration in the atmosphere, rising temperatures and extreme rainfall. These climate change induced disasters have exacerbated the risks of hunger and undernutrition [2] through the reduced yields of agricultural crops. Countries which already have food shortages, like low and middle income ones, are often the most vulnerable to climate change, and they have a low capacity to adapt [3].

Zimbabwe has experienced a number of severe drought episodes in the last three decades; 1991–1992, 1994–1995, 2002–2003, 2015–2016, and 2018–2019 [4]. In addition to droughts, the incidences and frequencies of cyclones have increased. Cyclone Japhet occurred in 2003, resulting in widespread river flooding causing extensive crop damage in Mozambique, Zambia, and Zimbabwe [5]. Cyclone Dineo followed in 2017 and then Cyclone Idai in 2019. After Cyclone Idai hit Zimbabwe, 6 weeks later Cyclone Kenneth hit the same areas giving no room for recovery in the affected zones [6].

In Zimbabwe, Cyclone Idai affected approximately 270,000 people of which about 51,000 were displaced [7]. This disaster claimed more than 340 lives and some people are still missing. Areas in Chimanimani and Chipinge districts suffered the greatest impact [7]. With agricultural land rendered unusable and infrastructure e.g., storage facilities left inutile; this possibly affected dietary intake and diet quality but however, this was not assessed [8]. Research has shown that cyclones not only destroy infrastructure but also wash away food stocks, granaries, fields, gardens, and livestock [9–11]. Climate change can accelerate undernutrition through several pathways, some of which include the disruption of household food security, child feeding practices, environmental health and access to health services [12]. An impact of these pathways results in food shortages, no access to safe and nutritious food, therefore, food consumption and dietary diversity are distorted. When a disaster occurs, women and children often suffer the most impact due to gender discriminatory cultural norms and inadequate access to resources [13]. Food shortages after a disaster render women more vulnerable to malnutrition because they have specific nutritional needs during adolescence, while pregnant and/or lactating women also tend to consume fewer calories to give priority to men and children [14]. Young children especially those below the age of five are often very sensitive to nutritional deficits. When children are exposed to such conditions of inadequacy especially during the first 1000 days, there are some irreversible damages that can occur to their cognitive development, health, and physical status. There is therefore a need to constantly check the nutritional health of these vulnerable groups through dietary assessments. Some of the dietary assessment methods include the use of 24-h dietary recalls (Household Dietary Diversity), food records and food frequency questionnaires [15]. Household dietary diversity (HDD) assesses the number of food groups consumed by a household over a stipulated period, and can be used as an indicator of food security as a higher HDDS value is correlated with caloric and protein adequacy [16]. The Food Consumption Score (FCS) not only considers dietary diversity and food frequency but also the relative nutritional importance of different food groups to give a better understanding of a household’s food security status. Minimum dietary diversity score is a population-level indicator of diet diversity that is used to validate micronutrient adequacy of the diet for both women of child bearing age and children under 5 years [15].

After Cyclone Idai hit Zimbabwe, approximately 3905 children between the ages of six and 59 months were admitted into treatment programmes for severe acute malnutrition. This showed the negative impact that Cyclone Idai had on child nutrition and food security. Also and relatedly, in 2020, Cyclone Amphan hit Bangladesh’s south western coast, resulting in instability on food security as well as economic instability [17]. Cyclone Amphan left 40.8% of the adults with severe food insecurity due to job loss or loss of income, and a decrease in the living conditions. Disruption of livelihoods further paved way for increased prevalence of child malnutrition due to decreased dietary quality [18].

Most studies on climate change induced natural disasters mainly look at impact of natural disasters on the environment, agricultural productivity, livelihoods and water, sanitation and hygiene. Few studies [19] have researched on the association of cyclones and food security outcomes. The majority of the studies done on natural disasters have looked at the effect of cyclones on agriculture output [20], livelihoods [21, 22], and poverty [23, 24]. Therefore, the main objective of this study was to assess the association between Cyclone Idai on selected food and nutrition security outcomes (Food Consumption Score, Households Dietary Diversity Score, Minimum Dietary Diversity Score for Women and Minimum Dietary Diversity Score for Children) in the most cyclone affected region of Eastern Zimbabwe. The authors selected these nutrition indicators because they reflect access to a variety of foods at the household and individual level. Natural disasters are known to greatly impact first access to foods which is partly the main mechanism by which they cause food insecurity.

## Methodology

### Data collection

#### Study setting

**Fig. 1:**
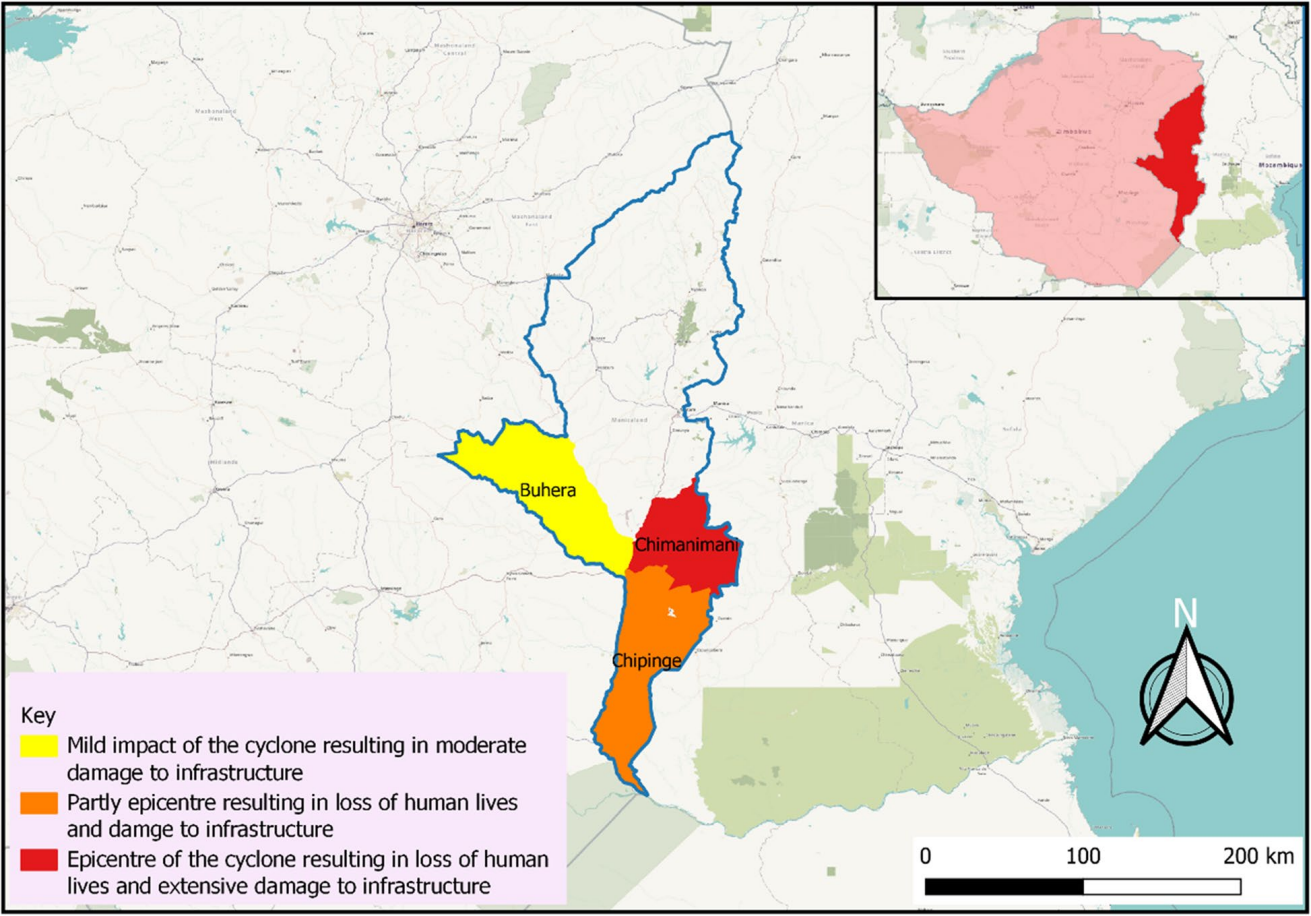
Map of Manicaland Province showing Buhera, Chimanimani and Chipinge Districts

The study was done in Eastern Zimbabwe, Manicaland Province, in the districts of Buhera, Chimanimani and Chipinge (Fig. 1). Manicaland is the second largest province in Zimbabwe with a population of 1,753,000 inhabitants [25]. The province is in the eastern most part of Zimbabwe (18.9216° S, 32.1746° E) and due to its proximity to Mozambique and the Indian Ocean it is prone to cyclones and other adverse weather events.

#### Sample size and data collection

Using the Dobson formula [26], a sample size of 418 households was calculated. We anticipated a high non-response rate due to high prevalence of temporary shelters, therefore, we included a non-response rate of 28% giving our final sample size to be 535. The households were purposively recruited based on the impact of Cyclone Idai through consultations with chiefs, headmen and key stakeholder meetings.

#### Data collection tools

Data was collected using face-to-face interviews. Enumeration was done with the help of 10 [9] trained enumerators in each district fluent in the local language. A questionnaire adopted from the Zimbabwe Vulnerability Assessment Committee [27] was used to collect household quantitative data. This questionnaire consisted of the following sections: Household demographics, 24 hour and 1-week dietary recall section for individual and household. Data was captured on an android-based software called Kobo toolbox, a free platform for collecting humanitarian and research data.

Nutrition indicators like household dietary diversity, food consumption scores, minimum dietary diversity for women and for children were calculated as indicated below.

##### Household dietary diversity score (HDDS)

Household dietary diversity (HDDS) is used to measure the quality of diet especially macro- and micronutrients. It depicts household access to a variety of food groups. HDDS as an indicator gives a better reflection of food security at household and intra-household levels. Data was collected using a 24-hr recall method. There are 12 food groups used to calculate the household dietary diversity score namely, (1) Cereals, (2) Roots and tubers, (3) Vegetables, (4) Fruits, (5) Meat, poultry, and offals, (6) Eggs, (7) Fish and seafood, (8) Pulses, legumes, and nuts, (9) Milk and milk products, (10) Oils/ fats, (11) Sugar/ honey and (12) Miscellaneous. A household is given a score if it consumed food from a food group listed above. The HDDS variable was calculated for each household. The value of this variable ranges from zero (0) to twelve (12).

##### Minimum dietary diversity score women (MDD-W)

The minimum dietary diversity score for women was measured according to the FAO guidelines for measuring minimum dietary diversity score for women [28]. It measures micronutrient adequacy in the diets of women at the population level. All the foods consumed by women of reproductive age (15–49 years) at or outside the home during the previous day or night (last 24 hours) was recorded. To compute the score, the foods were assigned into the following 10 food groups: (1) Grains, roots, and tubers, (2) Pulses, (3) Nuts and seeds, (4) Dairy, (5) Meat, poultry, and fish, (6) Eggs, (7) Dark leafy greens and vegetables, (8) Other Vitamin A-rich fruits and Vegetables, (9) Other vegetables, (10) Other fruits. The threshold for adequacy is 5 or more food groups.

##### Minimum dietary diversity for children (MDD-C)

Minimum dietary diversity for children is defined as the proportion of children 6–23 months of age who receive foods from four or more food groups. It is calculated as:

$$Children\ 6-23\ months\ of\ age\ who\ received\ foods\ from\ge 4\ food\ groups\ during\ the\ previous\ day\div Children\ 6-23\ months\ of\ age$$

The 7 foods groups used for determination of this indicator are: (1) grains, roots, and tubers, (2) legumes and nuts, (3) dairy products (milk, yogurt, cheese), (4) flesh foods (meat, fish, poultry, and liver/organ meats), (5) eggs, (6) vitamin-A rich fruits and vegetables, (7) other fruits and vegetables. A cut off of at least four food groups is associated with better quality of diets.

##### Food Consumption score (FCS)

Food consumption data was used to calculate food consumption scores consistent with the WFP methodology [29]. The food consumption score (FCS) was measured by collecting both consumption and frequency of different food groups by a household during the past 7 days before the survey. To calculate the FCS, standard weights were attached for each of the food groups that comprise the food consumption score. The food consumption groups include: starches, pulses, vegetables, fruit, meat, dairy, fats, and sugar. The consumption frequencies of the different foods in the groups were summed, with the maximum value for the groups capped at 7. The formula, based on these groups, with the standard weights, is: FCS = (starches*2) + (pulses*3) + vegetables + fruit + (meat*4) + (dairy*4) + (fats*.5) + (sugar*.5) (Oils*.5). The food consumption score therefore ranges from 0 to 112. FCS values from zero (0) to 28 indicates a poor FCS, 28.5 to 42 indicates a borderline FCS and from 35.5 to 112 indicates an acceptable FCS.

##### Severity of cyclone Idai

The severity of Cyclone Idai was grouped into five (5) categories which are: (i) not affected, (ii) moderately affected, (iii) extensively affected but still living in their homes, (iv) extensively affected, and relocated to camps, and (v) extensively affected and relocated to new houses. This categorization was based on the extent of damage to infrastructure (including shelter) and loss of human lives due to the cyclone. In the absence of a published scale, we developed our own severity scale basing on damage to infrastructure and loss of lives according to a similar scale by Caldera et al., 2016 and Caldera & Wirasinghe, 2015 whereby they categorized the severity of natural disasters based on the extent of damage to infrastructure, environment, fatalities and injuries sustained.

### Data analysis

Data was downloaded from Kobo toolbox cloud storage and exported from Microsoft excel to SPSS v20 (Microsoft Inc., Chicago Illinois). Data cleaning and coding was done in SPSS v20.

Linearity of continuous variables was tested using QQ plots. For demographics, frequency tables were generated. Association between minimum dietary diversity for women, household dietary diversity, food consumption scores and severity were tested using Pearson Correlation test (continuous variables) and Chi square (categorical variables) where appropriate, with significance set at *p* < 0.05. Linear regression analysis was done to test for determinants of HDDS and FCS, and logistic regression for MDD-W.

The study was conducted according to the guidelines of the Declaration of Helsinki and approved by the Research Ethics Committee of Marondera University of Agricultural Sciences and Technology (MUAST-26/22).

## Results

### Background characteristics

A total of 535 households consisting of 171 women of reproductive age and 213 children were interviewed (Table 1). The mean age for the household head was 45.9(40.14) years and the mean household size was 5.7(2.9). The youngest head of household was 12 years, with the oldest at 92 years old. The number of persons per household varied from one (1) to 27 members. Of the 535 households interviewed, the majority (334 households) were married and living together, with 48.2% of these still living in their homes after extensive damage by Cyclone Idai. Most of the households were male headed (84.7%). The majority of the household heads who were formally employed were in Category 3 (3.9%) whilst 100% of household heads in Category 4 had informal employment. In addition, informal employment was high in all Categories (> 50%).

**Table 1:** Household demographics

Variable	Characteristic	Severity of cyclone Idai (%)					***P***-value*
		Not affected	Moderately affected	Extensive but still living in their homes	Extensive (relocated to camps	Extensive (relocated to new houses)	
District	Buhera (*n* = 231)	39.8	34.6	25.5	0	0	0.000
	Chimanimani (*n* = 125)	0	0	77.6	8.0	14.4	
	Chipinge (*n* = 170)	0	40.6	59.5	0	0	
Type of settlement	Village (*n* = 461)	20	30.4	45.8	0	3.9	0.000
	Camp (*n* = 10)	0	0	0	100	0	
	Other (*n* = 55)	0	16.4	83.6	0	0	
Age of Household Head	Mean (*n* = 526)	47.4 (16.4)	49.9 (71.0)	44 (15.9)	49.3 (13.4)	43.8 (17)	0.690
Size of Household	Mean (*n* = 526)	5.7 (3.3)	6.2 (2.9)	5.7 (2.7)	5.5 (2.2)	3.9 (1.8)	0.030
Sex of Household head	Male (*n* = 205)	87.5	80.6	86.1	100	75	0.675
	Female (*n* = 37)	12.5	19.4	13.9	0	25	
Employment Status	Formal employment	1.1	1.4	3.9	0	0	0.024
	Informal employment	87	88.4	75.2	100	72.2	
	^a^Not categorised	12	10.2	20.9	0	27.8	
Marital Status	Married living together (*n* = 334)	20.1	27.2	48.2	0.9	3.6	0.005
	Married living apart (*n* = 35)	14.3	31.4	45.7	8.6	0	
	Divorced/Separated (*n* = 41)	7.3	31.7	53.7	4.9	2.4	
	Widow/Widower (*n* = 84)	19	35.7	41.7	1.2	2.4	
	Never married (*n* = 21)	0	9.5	71.4	4.8	14.3	
	Other (*n* = 11)	9.1	18.2	72.7	0	0	
Highest level of education of household head	None (*n* = 51)	3.9	39.2	54.9	0	2.0	0.007
	Primary (*n* = 185)	18.4	30.8	44.3	3.2	3.2	
	ZJC (*n* = 84)	23.8	36.9	33.3	4.8	1.2	
	O′ level (*n* = 164)	17.1	18.3	60.4	0	4.3	
	A’ level (*n* = 6)	16.7	0	83.3	0	0	
	Diploma/certificate after primary (*n* = 1)	0	0	100	0	0	
	Diploma/certificate after secondary (*n* = 7)	0	42.9	42.9	0	14.3	
	Graduate/post-graduate (*n* = 1)	100	0	0	0	0	
	Other *n* = 27	22.6	29.6	40.7	0	7.4	

* Chi Square for comparing categorical variables and ANOVA for comparing means

^a^ Not categorised group did not specify their employment status

There was a significant between severity of cyclone and district (*χ*^*2*^ = 305.700; df = 8; *p* = 0.000). Chimanimani was the most affected district with 77.6% being extensively affected but still living in the same homes. In addition, there was a significant association between severity and type of settlement (*χ*^*2*^ = 557.201; df = 8; *p* = 0.000). Furthermore, the results in Table 1 show a significant association between severity of Cyclone Idai and marital status (*χ*^*2*^ = 40.274; df = 20; *p* = 0.005) and severity of Cyclone Idai and education level of household head (*χ*^*2*^ = 55.123; df = 32; *p* = 0.007). The results also reveal a significant difference in household size across severity category (*p* = 0.030). The households worst affected and requiring relocation were the smallest in size (3.9(1.8)). However, there was no significant difference in age of household head across severity (*p* = 0.690) as well as gender of household head (*p* = 0.675).

### Association between cyclone Idai and nutrition outcomes

#### Food consumption score (FCS)

The overall median FCS was 32 [21.50, 45.80]. In addition, the median FCS for Category 1 (not affected) was 32 [22.00, 46.00], Category 2 was 30.5 [12.50, 40.50], Category 3 was 35 [22.25, 52.75], Category 4 was 10 [7.00, 24.00] and Category 5 was 27.75 [20.38, 37.88] (Table 2). The proportion with a poor FCS was 16, 27.2, 46.4, 6.4 and 4.0% for category 1 to 5 respectively. There was a significant association between the severity of Cyclone Idai and FCS (*χ*^*2*^ = 27.421; df = 8; *p* = 0.001). The highest proportion of households with a poor FCs were found in the first 3 categories (not affected, moderate and extensive but still living in their homes).

**Table 2:** Nutrition outcomes of study population by severity of Cyclone Idai^a^

Nutrition outcome	Characteristic	Severity of Cyclone Idai (%)					***P***-value*
		Not affected	Moderately affected	Extensive (Still living in their homes)	Extensive (Relocated to camps)	Extensive (Relocated to new houses)	
Food Consumption Score	Poor (*n* = 125)	16.0	27.2	46.4	6.4	4.0	0.001
	Borderline (*n* = 165)	18.8	33.3	41.8	1.2	4.8	
	Adequate (*n* = 217)	18.0	23.0	56.7	0	2.3	
HDDS	Mean (*n* = 500)	5.20 (1.4)	5.86 (2.0)	6.35 (2.0)	5.22 (1.2)	5.50 (2.0)	0.000
MDD-W	Inadequate (*n* = 102)	34.3	22.5	40.2	2.0	1.0	0.011
	Adequate (*n* = 60)	16.7	15.0	58.3	3.3	6.7	
MDD-C	Below average (*n* = 157)	25.5	25.5	42.0	3.8	3.2	0.245
	Above average (*n* = 49)	14.3	26.5	55.1	0	4.1	

*Pearson Chi square except where cells count was less than 5 Fisher’s exact was used

^a^All results show % proportion of households/individuals

#### Household dietary diversity score (HDDS)

HDDS was generally high (≥5 food groups) in all 5 categories of severity with Category 1 households having a HDDS of 5.20(1.4), Category 2 at 5.86(2.0), Category 3 at 6.35(2.0) and Category 4 and 5 at 5.22(1.2) and 5.50(2.0) respectively. There was a significant difference in HDDS across severity categories (*p* = 0.000).

#### Women minimum dietary diversity score (MDD-W)

The proportion of women below the MDD-W cut-off was as follows; 34.3, 22.5, 40.2, 2.0 and 1.0% for category 1–5 respectively. This proportion was highest for category 1 to 3. There was a significant association between MDD-W and severity of Cyclone Idai (*χ*^*2*^ = 12.220; df = 4; *p* = 0.016).

#### Children minimum dietary diversity score (MDD-C)

A high proportion of children in overall were below MDD-C cut-off (that is 74.1% of the children were below the cut-off for child dietary diversity). The proportion below the MDD-C cut-off by severity of cyclone was 25.5, 25.5, 42.0, 3.8 and 3.2% from category 1 to 5. There was however no significant difference in proportion below cut-off across severity category (χ^*2*^ = 5.439; df = 4; *p* = 0.245).

### Determinants of nutrition outcomes of sampled households

The results presented in Table 3 reveal that gender of household head was a negative predictor of HDDS (β = − 0.734, *p* = 0.040) which shows that female headed households were found to be more food secure. Marital status was a significant positive predictor of FCS (β = 0.093, *p* = 0.016) showing that married couples who are living together had a higher FCS than others. However, severity of Cyclone Idai was not a predictor of any of the nutrition outcomes (HDDS, FCS and MDD-W).

**Table 3:** Determinants of nutrition outcomes^a^

Variables	Nutrition Outcomes		
	HDDS	FCS	MDD-W
Age of household head	0.004	0.003	0.033
Marital status	0.145	0.093*	0.109
Sex of household head	−0.734*	−0.264	−0.593
Household size	0.067	−0.034	−0.057
Highest level of education	0.006	0.057	0.140
Type of settlement	−0.054	−0.087	0.699
Severity of Cyclone Idai	−0.315	− 0.100	0.613
Adjusted R^2^	0.103	0.042	
R^2^	0.134	0.075	

*Significant (*p* < 0.05)

^a^Simple linear regression with dependent variables, HDDS and FCS except for MDD-W where binary logistic regression was used

### Nutritional quality by severity of cyclone Idai

**Fig. 2:**
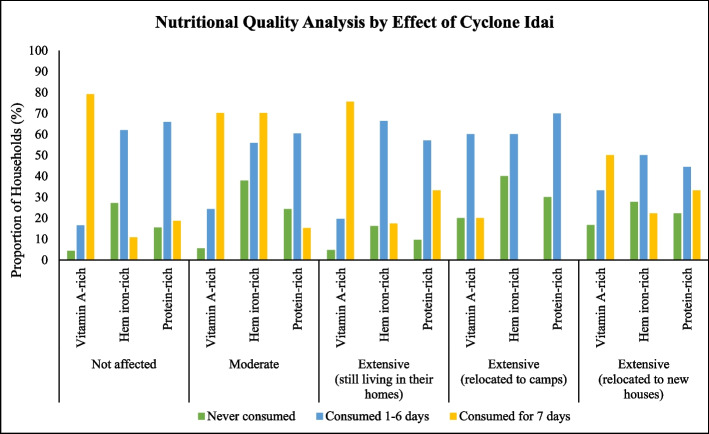
Nutritional Quality analysis by severity of Cyclone Idai

Consumption of Vitamin A-rich foods was in overall high in all settlements with only those living in camps (Category 4) having less than 50% households consuming Vitamin A-rich foods (Fig. 2). The highest proportion of households who never consumed these foods was also in camps. Households living in camps had the highest proportion (30%) of never consuming protein-rich foods; however, it also had the highest proportion of households who consumed protein-rich foods at least on 6 or less days prior to the survey. The proportion of households consuming Heme iron-rich foods daily is generally low across all the settlements. Camps had the highest proportion (40%) of households who never consumed Heme iron-rich foods seven (7) days before the survey and none of the households consumed Heme-iron foods daily.

## Discussion

This study sought to investigate the effect of Cyclone Idai on selected food security indicators namely FCS, HDDS, MDD-W and MDD-C. In general, the households worst affected by the cyclone were the smallest in size and headed by either divorced or never married household heads. Furthermore, households with poor socio-demographic profiles were more severely affected by the cyclone and this could be the reason they opted to be relocated since these households usually have poorer coping strategies and or support structures [30]. This is corroborated by findings from a study done in rural Sri Lanka [31] which showed that households that mostly depend on natural resources for their livelihood, and those with low incomes suffer greater losses from floods and droughts than other households. In terms of food security, contrary to our assertion that relocated households would have a poor FCS, the highest proportion of households with a poor FCs were found in the first 3 categories (not affected, moderate and extensive but still living in their homes). These findings corroborate with the ZimVAC 2019 report which showed that in Manicaland 30% of the surveyed households had poor FCS in 2019 which was after the cyclone had occurred as compared to the ZimVAC of 2018 which had19% of the surveyed households who had a poor FCS [32]. In general, these are food insecure districts, although relocated households had higher FCS possibly due to food aid. Data collection was conducted 31 months after the cyclone and by this time camps had been set up and there was relief activity by the Ministry of Health and Child Care alongside various Non-Governmental Organisations like the Adventist Development and Relief Agency International (ADRA), IOM, UNICEF, Nutrition Action Zimbabwe (NAZ), Save the Children, World Vision International, GOAL and WFP. IOM was working to support affected communities through technical assistance in shelter and information management.

The diet quality for women in the severely affected categories appeared to have been better than that of women in moderately and non-affected households. The proportion of women below the MDD-W cut-off was highest for category 1 to 3 and lowest for relocated households (severely affected). This validates the important contribution of food aid as part of relief efforts. To support our results, a study done in Southern African countries also showed that increased frequencies of flooding were associated with decreased women dietary diversity and food security [33].

Though HDDS was adequate across all categories, the results revealed that a high proportion of children were below MDD-C cut-off (≥4 food groups). This reflects access by households to food but poor intra-household food distribution. Our findings can be supported by results from a study done by Fanzo et al. [12] showed that climate change is associated with nutrition status through decreased food quantity and access individually, at household level or even at national level leading to a decrease in dietary diversity. However, the last 2 categories (relocated to camps or new homes) had the lowest proportion, meaning children living in relocated households (severely affected) had better access to diverse foods than those living in moderately and non-affected areas. This result contradicts with the types of food groups reportedly consumed. We found that the highest proportion of households who never consumed vitamin A rich foods were in the camps. Camps also had the highest proportion (40%) of households who never consumed Heme iron-rich foods. According to ZimVAC (2019), in Manicaland Province only 6% of the surveyed households had consumed iron-rich foods for over 6 days in the week the survey was done. This shows that consumption of iron-rich foods is basically low in these districts. Further, households living in camps had the highest proportion (30%) of never consuming protein-rich foods. Households in camps were entirely dependent on food aid. The food aid ration consisted of 2.4 kg pulses, 13.5 kg cereal, 0.75 kg vegetable oil, 6 kg super cereal plus (Corn Soya Blend++) and 6 kg super cereal (Corn Soya Blend) per person per month. It is evident that the food aid basket is lacking in animal source foods and perhaps the participants may not have known that the super cereal provided was fortified with Vitamin A hence it was a vitamin A source. Super cereal is prepared from heat treated maize, whole soya beans, vitamins and minerals to provide a highly nutritious meal [34]. A study done in Malawi showed that the provision of super cereal increased calorie density as well as enhancing the absorption of fat-soluble vitamins, thereby addressing the nutritional needs of children with moderate acute malnutrition [35].

Our study had several strengths which include that it had a big sample size, the findings presented in this paper provide a good baseline to inform future programming of food aid activities during disasters and it was the first study to attempt to associate cyclone disasters with selected nutrition indicators. More so, our findings call for evidence-based policies regarding composition of a food aid basket and targeting of beneficiaries. However, the main limitations of our study were that we could not gain access to communities immediately after the cyclone, hence the relationships measured were confounded by relief activities. We also did not collect nutrition status data and background socio economic status of the population which is an important confounder affecting coping strategies therefore we recommend this for future studies.

## Conclusion

The cyclone negatively affected most food security indicators with only the relocated households faring better possibly due to aid. There were variations in diet quality. Though at household level dietary diversity was adequate, this did not translate to adequate women and child dietary diversity. Food security indicators for children were below acceptable levels indicating inadequacy of relief activities for children and women dietary requirements. These findings call for evidence-based policies regarding composition of a food aid basket and targeting of beneficiaries.

## Data Availability

The data presented in this study are available on request from the corresponding author, upon signing of data use agreement and Research Ethics Committee approval.

## References

[1] Thomas V, LLpez R. Global increase in climate-related disasters. SSRN Journal [Internet] 2015; Available from: http://www.ssrn.com/abstract=2709331. [Cited 2022 Mar 21].

[2] Tirado MC, Hunnes D, Cohen MJ, Lartey A (2015). Climate change and nutrition in Africa. Journal of Hunger & Environmental Nutrition.

[3] Tirado MC, Crahay P, Mahy L, Zanev C, Neira M, Msangi S (2013). Climate change and nutrition: creating a climate for nutrition security. Food Nutr Bull.

[4] Frischen J, Meza I, Rupp D, Wietler K, Hagenlocher M (2020). Drought risk to agricultural Systems in Zimbabwe: a spatial analysis of Hazard, exposure, and vulnerability. Sustainability..

[5] Mukwenha S, Dzinamarira T, Chingombe I, Mapingure MP, Musuka G (2021). Health emergency and disaster risk management: a case of Zimbabwe’s preparedness and response to cyclones and tropical storms: we are not there yet!. Public Health in Practice.

[6] UNICEF. Massive flooding in Mozambique, Malawi and Zimbabwe [Internet]. 2020. Available from: https://www.unicef.org/stories/massive-flooding-malawi-mozambique-and-zimbabwe. [Cited 2022 Jun 18].

[7] Chatiza K (2019). Cyclone Idai in Zimbabwe: an analysis of policy implications for post-disaster institutional development to strengthen disaster risk management.

[8] Guterres A. “Mozambique, Zimbabwe and Malawi have suffered one of the worst weather-related catastrophes in the history of Africa” [Internet]. [Cited 2022 April 20] Available from: http://www.xinhuanet.com/english/2019-03/27/c_137925714.htm.

[9] Chapagain T, Raizada MN (2017). Impacts of natural disasters on smallholder farmers: gaps and recommendations. Agriculture & Food Security.

[10] OCHA. 2019 Zimbabwe Flash Appeal, January–June 2019 (Revised following Cyclone Idai, March 2019) - Zimbabwe [Internet]. ReliefWeb. 2019. Available from: https://reliefweb.int/report/zimbabwe/2019-zimbabwe-flash-appeal-january-june-2019-revised-following-cyclone-idai-march. [Cited 2022 Mar 28].

[11] Dembedza VP, Chopera P, Mapara J, et al. Impact of climate change-induced natural disasters on intangible cultural heritage related to food: a review. J Ethn Food. 2022;9:32. 10.1186/s42779-022-00147-2.

[12] Fanzo J, Davis C, McLaren R, Choufani J (2018). The effect of climate change across food systems: implications for nutrition outcomes. Global Food Security.

[13] Neumayer E, Plümper T (2007). The gendered nature of natural disasters: the impact of catastrophic events on the gender gap in life expectancy, 1981–2002. Ann Assoc Am Geogr.

[14] Chingarande D, Mugano G, Chagwiza G, Hungwe M. Zimbabwe Market Study: Manicaland Province Report [Internet]. [Cited 2022 April 28] Available from: https://www.rtachesn.org/wp-content/uploads/2020/01/RTAC-Zimbabwe_Market-Study_Manicaland-Province-1.pdf.

[15] Picó C, Serra F, Rodríguez AM, Keijer J, Palou A (2019). Biomarkers of nutrition and health: new tools for new approaches. Nutrients..

[16] Fiedler JL, Lividini K, Bermudez OI, Smitz MF (2012). Household consumption and expenditures surveys (HCES): a primer for food and nutrition analysts in low- and middle-income countries. Food Nutr Bull.

[17] Hossain A, Ahmed B, Rahman T, Sammonds P, Zaman S, Benzadid S (2021). Household food insecurity, income loss, and symptoms of psychological distress among adults following the cyclone Amphan in coastal Bangladesh. PLoS One.

[18] Hossain M, Uddin M, Rokanuzzaman M, Miah M, Alauddin M. Effects of Flooding on Socio-Economic Status of Two Integrated Char Lands of Jamuna River, Bangladesh. J Environ Sci Nat Resour. 2015;6(2):37–41. 10.3329/jesnr.v6i2.22093.

[19] Paul SK, Paul BK, Routray JK (2012). Post-cyclone Sidr nutritional status of women and children in coastal Bangladesh: an empirical study. Nat Hazards.

[20] Chikodzi D, Nhamo G, Chibvuma J. Impacts of tropical cyclone Idai on cash crops agriculture in Zimbabwe. In: Nhamo G, Chikodzi D, editors. Cyclones in southern Africa: volume 3: implications for the sustainable development goals [internet]. Cham: springer international publishing; 2021 [cited 2022 Jun 9]. p. 19–34. (sustainable development goals series). 10.1007/978-3-030-74303-1_2.

[21] Solayman HM. Impacts of cyclone on livelihood: Study on a coastal community. Int J Nat Soc Sci. 2017;4(4):56-64.

[22] WFP. Threat to lives and livelihoods as cyclone Batsirai hurls towards Madagascar | World Food Programme [Internet]. 2022. Available from: https://www.wfp.org/news/threat-lives-and-livelihoods-cyclone-batsirai-hurls-towards-madagascar. [Cited 2022 Jun 9].

[23] Ishizawa OA, Miranda JJ (2016). Weathering storms: understanding the impact of natural disasters on the poor in Central America.

[24] Warr P, Aung LL (2019). Poverty and inequality impact of a natural disaster: Myanmar’s 2008 cyclone Nargis. World Dev.

[25] ZimStat POB. Zimbabwe Population Census. 2012;152 [Internet]. [Cited 2022 March 28] Available from https://www.zimstat.co.zw/wp-content/uploads/publications/Population/population/census-2012-national-report.pdf.

[26] Naing L, Winn TB, Rusli BN. Practical issues in calculating the sample size for prevalence studies. Arch Orofacial Sci. 2006;1:9-14.

[27] ZimVAC. Zimbabwe Vulnerability Assessment Committee (ZimVAC). 2020;97 [Internet]. [Cited 2022 Nov 9] Available from: https://fnc.org.zw/wp-content/uploads/2020/10/Zimbabwe-Vulnerability-Assessment-Committee-2020-Rural-Livelihoods-Assessment-Report.pdf.

[28] FAO. Minimum dietary diversity for women: An updated guide to measurement - from collection to action [Internet]. Rome, Italy: FAO; 2021. 176 p. Available from: https://www.fao.org/documents/card/en/c/cb3434en. [Cited 2022 Mar 28].

[29] wfp197216.pdf [Internet]. Available from: https://documents.wfp.org/stellent/groups/public/documents/manual_guide_proced/wfp197216.pdf. [Cited 2022 Mar 28].

[30] Hallegatte S, Vogt-Schilb A, Rozenberg J, Bangalore M, Beaudet C (2020). From poverty to disaster and Back: a review of the literature. EconDisCliCha..

[31] De Silva MMGT, Kawasaki A (2018). Socioeconomic vulnerability to disaster risk: a case study of flood and drought impact in a rural Sri Lankan community. Ecol Econ.

[32] ZimVAC 2019 Rural Livelihoods Assessment Report | HumanitarianResponse [Internet]. Available from: https://www.humanitarianresponse.info/en/operations/zimbabwe/assessment/zimvac-2019-rural-livelihoods-assessment-report. [Cited 2022 Nov 9].

[33] Niles MT, Emery BF, Wiltshire S, Brown ME, Fisher B, Ricketts TH (2021). Climate impacts associated with reduced diet diversity in children across nineteen countries. Environ Res Lett.

[34] wfp251114.pdf [Internet]. Available from: https://documents.wfp.org/stellent/groups/public/documents/manual_guide_proced/wfp251114.pdf. [Cited 2022 Jun 18].

[35] Langlois B, Suri D, Wilner L, Walton S, Chui K, Caiafa K (2017). Self-report vs. direct measures for assessing corn soy blend porridge preparation and feeding behavior in a moderate acute malnutrition treatment program in southern Malawi. Journal of Hunger & Environmental Nutrition..

